# Late, but not early, arriving younger siblings foster firstborns’ understanding of second-order false belief

**DOI:** 10.1016/j.jecp.2017.08.007

**Published:** 2018-02

**Authors:** Amy L. Paine, Holly Pearce, Stephanie H.M. van Goozen, Leo M.J. de Sonneville, Dale F. Hay

**Affiliations:** a Cardiff University, Cardiff CF10 3AT, UK; b Leiden University, 2311 EZ Leiden, The Netherlands

**Keywords:** Theory of mind, Siblings, Second-order false belief, Social cognition, Longitudinal study, Community sample

## Abstract

This study examined the influence of younger siblings on children’s understanding of second-order false belief. In a representative community sample of firstborn children (*N* = 229) with a mean age of 7 years (*SD* = 4.58), false belief was assessed during a home visit using an adaptation of a well-established second-order false belief narrative enacted with Playmobil figures. Children’s responses were coded to establish performance on second-order false belief questions. When controlling for verbal IQ and age, the existence of a younger sibling predicted a twofold advantage in children’s second-order false belief performance, yet this was the case only for firstborns who experienced the arrival of a sibling after their second birthday. These findings provide a foundation for future research on family influences on social cognition.

## Highlights

• Few studies have examined the influence of siblings on theory of mind in middle childhood.
• A second-order false belief task enacted with Playmobil® figures was given to 229 7-year-olds.
• Children with younger siblings outperformed those without.
• However, children with early arriving younger siblings did not have the same advantage.
• These findings demonstrate younger siblings can foster theory of mind beyond the preschool years.

## Introduction

Individual differences in children’s development of *theory of mind* (ToM), defined as the “understanding of mental states, what we know or believe about thoughts, desires, emotions, and other psychological entities both in ourselves and in others” (Miller, 2009, p. 749), have traditionally been explored using the *false belief task* (Perner & Wimmer, 1983). Researchers have noted various sources of individual differences on this task, including number of siblings (Lewis et al., 1996, McAlister and Peterson, 2007, Perner et al., 1994, Ruffman et al., 1998), family sociodemographic status (Cole and Mitchell, 2000, Cutting and Dunn, 1999), and maternal education level (Pears & Moses, 2003). Passing false belief tasks has also been found to be related to children’s language (Astington & Jenkins, 1999) and executive function (Carlson & Moses, 2001).

Although it is well established that preschoolers with older siblings outperform those without siblings on ToM tasks (Lewis et al., 1996, Ruffman et al., 1998), the influence of younger siblings on ToM remains unclear. Piaget (1959) suggested that both younger and older siblings facilitate social understanding through discussion and reflection. Dunn (1994) claimed that siblings influence social understanding through talk about causality and internal states, management of conflict by parents, joint play, shared jokes, and reasoning about moral issues; both younger and older siblings may equally facilitate ToM (Jenkins and Astington, 1996, Perner et al., 1994, Peterson, 2000). Indeed, in a recent meta-analysis by Devine and Hughes (2016), the number of child-aged siblings, regardless of birth order, predicted false belief understanding during early childhood.

Despite these findings, the evidence is mixed. Some studies found no effect of younger siblings on ToM tasks (Calero et al., 2013, Farhadian et al., 2010, Ruffman et al., 1998, Shahaeian, 2015), and in one case younger siblings had a negative effect on ToM (Wright & Mahford, 2012). Younger siblings may influence ToM development negatively by placing increased demands on parents’ time, resulting in a decrease in mother–firstborn positive interactions (Baydar, Greek, & Brooks-Gunn, 1997), including play and conversation with the firstborn child (Dunn and Kendrick, 1980a, Dunn and Kendrick, 1980b). It is also possible that parents’ explanations to their firstborn children are frequently interrupted due to younger siblings’ demands (Wright & Mahford, 2012). The *age threshold model* proposes that younger siblings may need to reach a certain threshold in age before providing a positive influence on ToM (Kennedy, Lagattuta, & Sayfan, 2015). If so, it is possible that some null findings may be due to the younger siblings in those studies being too young to provide any advantage.

Younger siblings may become more important in fostering children’s more advanced understanding of minds during middle childhood, but research on sibling influences on the later development of ToM remains limited (Devine and Hughes, 2016, Hughes, 2016, Miller, 2009). Most studies examining younger sibling influence on ToM focused on first-order false belief tasks (Miller, 2009). However, during middle childhood, second-order false belief tasks are thought to be more age appropriate (Perner & Wimmer, 1985). Whereas first-order false belief tasks typically assess children’s understanding that someone may have beliefs that differ from their own, a second-order task assesses whether children understand that one story character can have a mistaken belief about another character’s belief. Some children pass this higher-order test of ToM between 6 and 7 years of age (Perner & Wimmer, 1985).

Findings about sibling influence on ToM in older children are mixed; in some cases both younger and older siblings facilitated higher-order ToM performance (Kennedy et al., 2015, McAlister and Peterson, 2007), but in other studies younger siblings had no effect (Calero et al., 2013, Miller, 2013). It is possible that older siblings begin to benefit from younger siblings as the latter become more proficient playmates (Lagattuta et al., 2015). Alternatively, as firstborn children start school and spend less time with family members, the initial sibling advantage may disappear.

Before a more definitive conclusion can be drawn, larger-scale studies are required to tease apart the benefits of particular kinds of sibling constellations (Cassidy, Fineberg, Brown & Perkins, 2005). Studies finding no effect of younger siblings may have lacked sufficient statistical power to detect smaller effects once samples are separated into *sibling constellation groups* (i.e., sibling presence, birth order, age spacing, and gender composition) (see Miller, 2013). “Only child” subsamples typically are small (Miller, 2013). This not only leads to a decrease in power to detect an advantage in having a sibling over none but also results in samples with a very high proportion of children who have siblings—in some studies more than 90%, which exceeds the estimate that 80% of children in Western families have a sibling (Volling, 2012).

Although previous research on ToM has highlighted covariates that need to be accounted for in studies of sibling influence, rarely have these all been controlled in a single study, which may also explain the mixed findings. These covariates include child age (Wellman, Cross, & Watson, 2001), family context (Lewis et al., 1996), sociodemographic risk factors (Cutting and Dunn, 1999, Cole and Mitchell, 2000), language ability (Astington & Jenkins, 1999), and executive function, specifically working memory and inhibition (Carlson and Moses, 2001, Lagattuta et al., 2014). Children’s understanding of second-order false belief is positively associated with their language and executive function (Astington et al., 2002, Lagattuta et al., 2010, Lagattuta et al., 2014, Perner et al., 2002). However, in studies of sibling influences on ToM, rarely are age, sociodemographic risk, language, and executive function all controlled (see Kennedy et al., 2015, Miller, 2013); when examined together in one study, executive function was positively associated with second-order false belief when age was controlled but not when language ability was partialed out (Hasselhorn, Mahler, & Grube, 2005).

Although correlates of first-order false belief may also be relevant for second-order false belief, this has not yet been fully established (Miller, 2012). Some of these correlates, such as executive function, may be most important during early development of ToM; after children reach a certain threshold of ToM skills during middle childhood, these relationships may attenuate or disappear (Lagattuta et al., 2015).

To address these issues, we explored the ways in which younger siblings might influence 7-year-olds’ performance on a second-order false belief task while controlling for known correlates of ToM in a study of a nationally representative community sample of firstborn children and their families. Our moderately sized dataset of firstborn children and their families provided a unique opportunity to examine the effect of younger sibling constellation factors, including sibling presence, gender composition, and age spacing.

## Method

### Design

The Cardiff Child Development Study (CCDS) is a prospective longitudinal study of a nationally representative sample of mothers and their firstborn children. Data collection took place during pregnancy and at means of 6, 12, 21, 33 and 84 months postpartum. The current study focuses on the home visit that took place at a mean age of 84 months. The CCDS is funded by the Medical Research Council (MRC), and ethical approval was obtained for the procedures from the National Health Service (NHS) Multi-Centre Research Ethics Committee and the Cardiff University School of Psychology Research Ethics Committee.

### Participants

A total of 332 primiparous women and their partners were recruited between November 2005 and June 2008 from NHS antenatal clinics in hospitals and general practitioner (GP) surgeries in two Health Care Trusts in Wales, United Kingdom. The CCDS is nationally representative in terms of sociodemographic factors; it did not significantly differ from families with firstborn children in the large, nationally representative sample in the Millennium Cohort Study (see Hay et al., 2014).

**Fig. 1:**
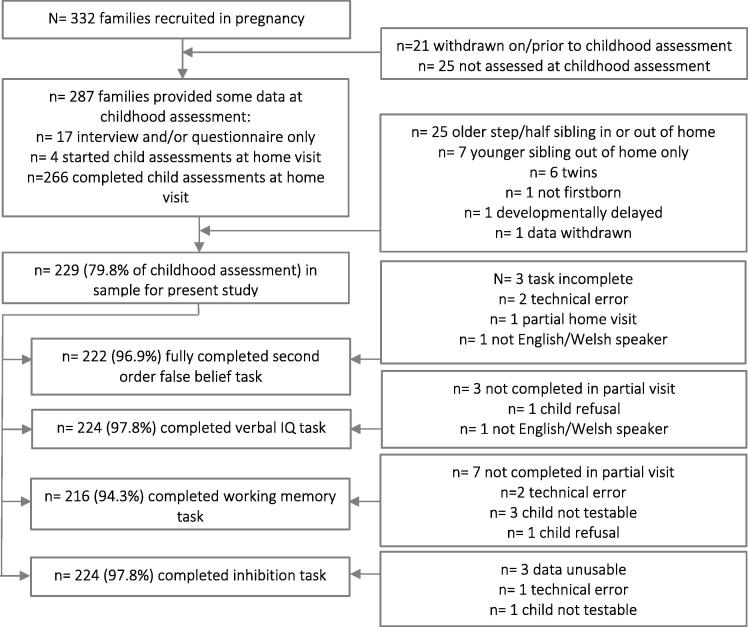
Derivation of the sample.

A total of 321 families were seen after the birth of the first child, with 286 (89.01%) assessed at 7 years; of these, 272 (95%) were directly observed at home. The current sample comprises 229 of these families (Fig. 1).

The participants’ mean age at the time of testing was 83.20 months (range = 67–104). The demographic characteristics of the children included in this subsample (69.0% of the original sample) are summarized in Table 1. A child’s exposure to socioeconomic adversity was indexed by (a) the mother not having achieved basic educational attainments (i.e., having no qualifications or fewer than five general certificates of secondary education (GCSEs) or equivalent attainments), (b) the mother being 19 years of age or younger at the time of the child’s birth, (c) the mother not being legally married during the pregnancy, (d) the mother not being in a stable couple relationship during the pregnancy, and (e) the mother’s occupation being classified as working class according to the Standard Occupational Classification 2000 (SOC2000; Elias, McKnight, & Kinshott, 1999). A principal components analysis (PCA) based on the polychoric correlation matrix confirmed that all of these items contributed to a single component, which explained approximately 77% of the shared variance in these risk indicators. Summary scores derived from this PCA measured the family’s exposure to socioeconomic adversity (Perra, Phillips, Fyfield, Waters, & Hay, 2015).

**Table 1:** Demographic information for total sample and subsample.

	Total sample	Subsample
	(*N* = 332)	(*N* = 229)
Mother’s age at first birth (mean years)	28.1	28.8
Social class (% middle class)	50.9	57.6
Mother’s education (% > basic qualifications)	78.3	81.6
Stable partnerships (% stable partnerships)	90.4	91.3
Legally married (% married)	50.3	57.2
Ethnicity (% British or Irish)	93.0	92.3
Sociodemographic adversity index (mean)	.00	−.13
Firstborn child gender (% female)	43.3	45.0

*Note.* The *N* = 229 in the current study was not significantly different from the original *N* = 332 recruited.

In terms of sibling constellation, 172 children (75.1%) had at least one younger sibling living in the home; of these, 133 (58.1%) had one sibling, 32 (14.0%) had two siblings, and 7 (3.1%) had three siblings. Gender composition and age spacing were examined with the younger sibling closest in age to the firstborn. In total, 91 children (52.9%) were in a same-gender sibling dyad and 81 children (47.1%) were in an opposite-gender sibling dyad; of these, there were 47 (27.3%) older boy–younger boy dyads, 44 (25.6%) older girl–younger girl dyads; 46 (26.7%) older boy–younger girl dyads, and 35 (20.3%) older girl–younger boy dyads.

The firstborn children entered siblinghood at a mean age of 35.7 months (*SD* = 16.8). To investigate the influence of sibling birth interval, children were grouped according to the interval between the firstborn and secondborn sibling births. Children who entered siblinghood at or below the first quartile (≤24 months) were categorized as having an *early arrival sibling* (*n* = 45, 19.7%), children who entered siblinghood at or above the third quartile (≥43 months) were categorized as having a *later arrival sibling* (*n* = 44, 19.2%), and children with a sibling arriving between these quartiles were categorized as having an *average arrival sibling* (*n* = 83, 36.2%).

### Procedure

Research assistants visited each family at home for two 2-h sessions. The caregiver (typically the mother) was given questionnaires and interviewed by a trained research assistant to gather information on the caregiver and firstborn’s well-being as well as family lifestyle arrangements and social network. Where possible, these interviews would take place in a separate room from the child. During these interviews, the child completed various cognitive, social, and emotional assessments in a quiet space with a second trained research assistant. A third research assistant attended to keep any younger siblings occupied while the assessments took place. A remuneration of £20 was given to the caregiver, and a book voucher of £10 was given to the child, at the end of the session.

### Measures

#### Second-order false belief task

**Fig. 2:**
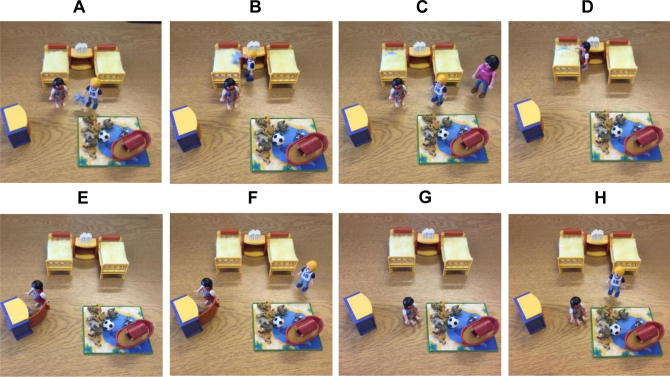
False belief story with Playmobil. In this illustration of the story, the protagonist (Nick) shows his special teddy to the child (A) and tucks the teddy inside the bed (B). The mother comes into the room and asks Nick to brush his teeth, and they leave the room (C). In Nick’s absence, the sibling removes the teddy from the duvet (D) and hides the teddy in the cupboard (E). Unbeknownst to Alex, Nick returns and watches Alex hiding the teddy (F) before leaving the room again (G). When Nick comes back into the room, he says, “I want my teddy.” (H).

This task was adapted from second-order belief paradigms (Coull et al., 2006, Perner and Wimmer, 1985). Each child was told a story enacted with plastic Playmobil figures by the experimenter. The protagonist was gender matched to the participant, and the sibling was gender matched to the participant’s closest-in-age younger sibling. In cases where the focal child had no siblings, the sibling character’s gender was randomly selected. The narrative is shown in Fig. 2.

**Fig. 3:**
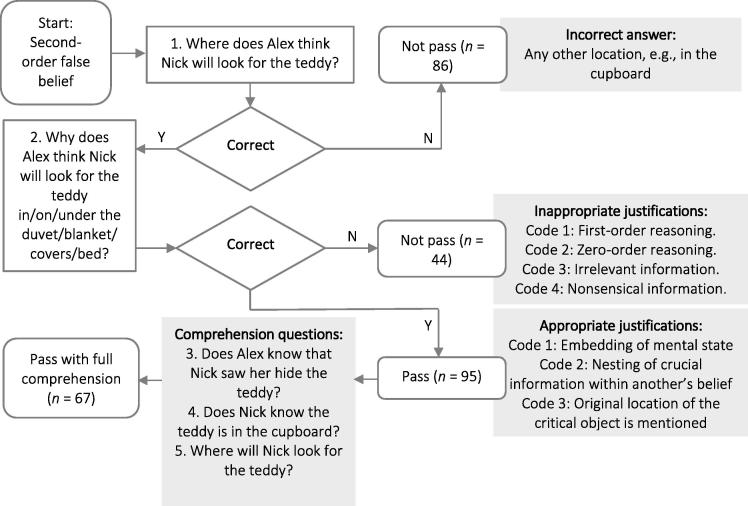
Flow diagram displaying pathways to passing and not passing second-order false belief in the false belief story.

Pathways to passing or not passing this task are shown in Fig. 3. Children were classified as minimally passing second-order false belief if they correctly answered the first location question with an appropriate justification, and they were classified as passing second-order false belief with full comprehension if they also correctly answered the additional probe questions. An independent observer coded transcripts for 32.9% of the participants and established excellent agreement for passing second-order false belief (kappa coefficient = 1.00) and for appropriate or inappropriate justifications (kappa coefficient = 1.00). There was also very good agreement within appropriate and inappropriate justification codes, where the kappa coefficients were .89 and .79, respectively.

#### Verbal IQ

Each child’s vocabulary knowledge was assessed using the British Picture Vocabulary Scale (BPVS; Dunn, Dunn, Whetton, & Pintillie, 1982). In this task, the experimenter spoke a word to the child, who was asked to point or say the number of the picture that corresponded to the word. Each child’s verbal IQ was calculated by age normalizing the data to produce a standardized score. The mean score for verbal IQ was 99.54 (*SD* = 11.99), and the average age children in the sample were equivalent to was 84.14 months (*SD* = 14.66) and ranged from 49 to 150 months.

#### Executive function

Cognitive function was assessed using tasks from the Amsterdam Neuropsychological Tasks (ANT) (de Sonneville, 1999). The ANT is a well-validated and sensitive instrument to evaluate executive functioning in population-based samples (Brunnekreef et al., 2007) and clinical samples (Rommelse et al., 2008). The tasks were presented on a laptop computer, and children made responses using a mouse. For each task, the experimenter gave verbal instructions while showing examples. Following this, children were given a practice trial before starting the test trials.

The Response Organization Objects (ROO) task was used to measure *response inhibition* via children’s reaction times to stimuli. Children were asked to hold the mouse with a forefinger of each hand on each button of the mouse. In Part 1 (compatible condition), children were presented with a fixation cross in the middle of the screen and were asked to respond to a red ball appearing on either side of the cross by clicking the same side of the mouse on which the ball appeared. In Part 2 (incompatible condition), children were presented with a white ball on the screen. Children were instructed to click the opposite side of the mouse according to the position of the ball. Response inhibition was operationalized as the difference between children’s mean reaction speed times in milliseconds (*M* = 314.32 ms, *SD* = 195.65) between the incompatible (Part 2) and compatible (Part 1) tasks.

The Visuo-Spatial Sequencing (VSS) task was used to measure visuo-spatial *working memory.* In this task, children were presented with a gray square containing 9 circles symmetrically positioned in a 3 × 3 matrix on a computer screen. After a beep, a sequence of circles was pointed at by a computer animated hand, and after the sequence children took control of the mouse to replicate the sequence of circles. The test consisted of 24 trials and gradually increased in difficulty in the number of targets and complexity of the sequence. Working memory was assessed using the total number of correct targets in the correct order, with a total of 100 possible correct targets. The mean score for correct targets in the correct order was 67.24 (*SD* = 17.94).

## Results

### Children’s understanding of second-order false belief

Correlations, means, and standard deviations for all variables of interest are presented in Table 2. In total, 95 children (42.8%) passed the minimal second-order false belief questions, and 67 children (30.2%) passed the second-order false belief questions with full comprehension (Fig. 3). Minimal second-order false belief and second-order false belief with full comprehension were positively associated (Table 2). A Guttman scaling analysis using the Goodenough–Edwards method revealed a developmental progression from minimal second-order false belief to second-order false belief with full comprehension (*C*_R_ = .99). Despite these two levels of passing second-order false belief being a part of a single continuum, younger sibling constellation factors were only associated with passing second-order false belief with full comprehension (Table 2). Therefore, the subsequent analyses focused on children’s full comprehension of second-order false belief. Prior to investigating the influence of siblings on this measure of false belief understanding, a preliminary investigation of its correlates was conducted.

**Table 2:** Intercorrelations among all variables of interest.

Variable	1	2	3	4	5	6	7	8	9	10	11
1. Presence of a sibling in the home	–										
2. Number of siblings living in the home	.77**	–									
3. Timing of sibling arrival	.^a^	−.33**	–								
4. Firstborn age at false belief tasks	.10	.03	.22**	–							
5. Firstborn gender	.03	.06	−.03	.01	–						
6. Second-order false belief minimal	.09	.07	−.03	.04	.10	–					
7. Second-order false belief full	.10*	.10	.02	.06	.12	.76**	–				
8. Sociodemographic risk	−.09	−.01	.15*	.25**	−.11	−.18**	−.18**	–			
9. Verbal IQ	−.01	−.05	−.12	−.23**	.07	.24**	.23**	−.47**	–		
10. Response inhibition	−.15*	−.15*	.13	−.12	.15*	−.07	−.04	−.07	−.01	–	
11. Working memory	−.02	.02	.07	.21**	.16*	.09	.09	−.24**	.32**	−.17*	–

Mean	0.75	0.95	35.68	83.20	0.45	0.43	0.30	−0.13	99.54	314.32	67.24
*SD*	0.43	0.71	16.84	4.59	0.50	0.50	0.46	0.97	11.99	195.65	17.94

*Note.* Associations between dichotomous variables were tested by kappa coefficients.

* *p* < .05.

** *p* < .001.

a Correlation not computed because one variable is constant.

### Correlates of second-order false belief understanding

Examination of the correlation matrix (Table 2) and the collinearity statistics revealed no issues with collinearity among predictor variables: firstborn age, firstborn gender, sociodemographic risk, verbal IQ, response inhibition ANT, and working memory ANT (variance inflation factor < 10, tolerance > .20) (Menard, 1995, Myers, 1990). Verbal IQ and sociodemographic risk were significantly associated with passing the second-order false belief questions with full comprehension, with higher verbal IQ scores associated with better performance and higher sociodemographic risk scores associated with lower performance.

No relationship was detected between the ANT measures of response inhibition and working memory and children’s passing second-order false belief with full comprehension, nor was a relationship detected between age at the time of testing and second-order false belief (all *p*s > .19) (Table 2). However, in view of earlier research suggesting that individual differences exist in performance on false belief tasks across different ages (Wellman et al., 2001), age was included in the subsequent logistic regression.

In the logistic regression, these potential confounds accounted for 11% of the variance in second-order false belief with full comprehension, *χ*^2^(3) = 18.45, *p* < .001, Nagelkerke *R*^2^ = .11. Children who were older at the time of testing, Wald statistic = 4.21, *p* < .05, odds ratio (*OR*) = 1.08, 95% confidence interval (*CI*) = 1.00–1.16, and those who had higher verbal IQ scores, Wald statistic = 7.17, *p* < .01, *OR* = 1.04, 95% *CI* = 1.01–1.07, performed significantly better on second-order false belief; therefore, age and verbal IQ were used as covariates in the subsequent analysis.

### Do younger sibling constellation factors influence the firstborn’s second-order false belief performance?

There was no significant association between number of siblings living in the home and second-order false belief performance (*r* = .10, *p* = .15) (Table 2). Therefore, all subsequent analyses explored sibling constellation factors related to the closest-in-age sibling.

#### Presence of a sibling in the home

**Fig. 4:**
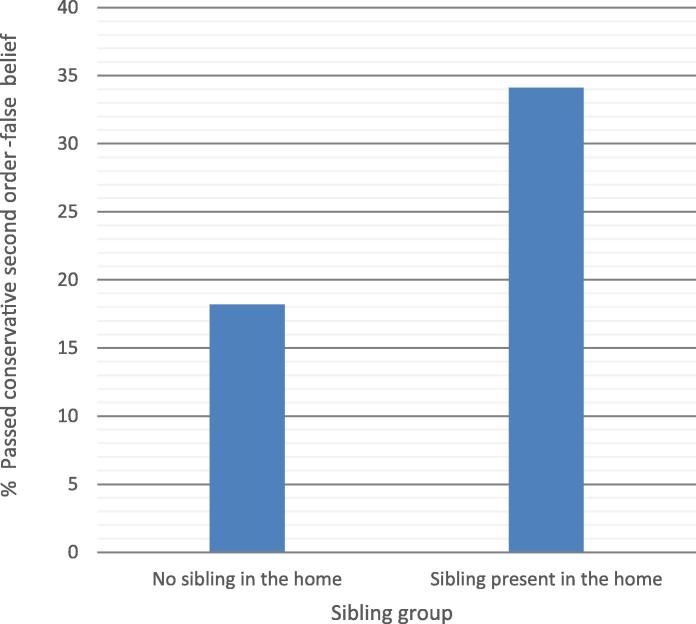
Percentages of children who passed second-order false belief with full comprehension according to whether the firstborn had a sibling present in the home.

To test for variations in second-order false belief as a function of presence or absence of siblings in the home, the sample was divided into two groups. Preliminary analyses showed no differences between the groups in ratio of boys to girls, firstborn mean age, sociodemographic risk, verbal IQ, and working memory (all *p*s > .15) (see Table 3). Children with siblings performed better on the response inhibition task, *t*(76.83) = 2.03, *p* < .05. Children with a sibling had a twofold advantage in passing the second-order false belief task with full comprehension, *χ*^2^(1) = 5.00, *p* < .05, *OR* = 2.33, 95% *CI* = 1.10–4.97 (see Fig. 4).

**Table 3:** Means and standard deviations of all variables of interest for sibling groups.

Variable	Sibling presence groups				Sibling arrival groups							
	No younger sibling present		Younger sibling present		Early arrival younger sibling		Average arrival younger sibling		Later arrival younger sibling		Average to later arrival younger sibling	
	Mean	SD	Mean	SD	Mean	SD	Mean	SD	Mean	SD	Mean	SD
Firstborn age at false belief tasks (months)	82.44	3.91	83.45	4.78	82.67	5.29	83.05	3.79	85.02	5.57	83.74	4.57
Firstborn gender	.42	.50	.46	.50	.42	.50	.48	.50	.45	.50	.47	.50
Second-order false belief	.33	.47	.46	.50	.39	.49	.55	.50	.37	.49	.49	.50
Second-order false belief full comprehension	.18	.39	.34	.48	.25	.44	.41	.50	.30	.46	.37	.49
Sociodemographic risk	.03	.95	−.19	.98	−.19	1.07	−.38	.82	.19	1.06	−.18	.95
Verbal IQ	99.78	12.54	99.46	11.85	99.18	11.91	101.37	11.92	96.23	11.18	99.56	11.88
Response inhibition	366.29	230.11	297.40	180.60	317.48	175.05	267.18	148.92	334.49	229.62	290.34	182.69
Working memory	67.73	18.50	67.09	17.82	64.20	19.44	69.29	16.12	66.00	18.93	68.14	17.15

In a subsequent logistic regression analysis (Table 4), the covariates were entered into the first step of the model, which accounted for 9% of the variance in second-order false belief understanding, *χ*^2^(2) = 15.07, *p* < .001, Nagelkerke *R*^2^ = .09. At the second step, the presence of a younger sibling accounted for significant additional variance in understanding second-order false belief, *χ*^2^(1) = 4.97, *p* < .05, and the overall model remained significant, *χ*^2^(3) = 19.98, *p* < .001, Nagelkerke *R*^2^ = .12. Within this model, verbal IQ remained a significant predictor of second-order false belief performance. Children with a younger sibling were twice as likely as children without siblings to pass second-order false belief with full comprehension, Wald statistic = 4.53, *p* < .05, *OR* = 2.35, 95% *CI* = 1.07–5.15.

**Table 4:** Logistic regression of presence of a younger sibling in the home, firstborn age, and verbal IQ as predictors of passing second-order false belief with full comprehension.

Variable	*R*^2^	*B*	*SE*	Wald *χ*^2^	OR (Odds Ratio)	95% *CI* for *OR*
Step 1	.09***					
Constant		−10.91	3.61	9.12	0.00	
Firstborn age		.06	.04	2.86	1.06	0.99–1.14
Verbal IQ		.05***	.01	12.68	1.05	1.02–1.08

Step 2	.12***					
Constant		−11.47	3.70	9.59	0.00	
Firstborn age		.06	.04	2.45	1.06	0.99–1.14
Verbal IQ		.05***	.02	13.00	1.05	1.02–1.08
Presence of a younger sibling		.85*	.40	4.53	2.35	1.07–5.15

*Note.* The table presents the total *R*^2^ Nagelkerke statistic. *N* = 219.

* *p* < .05.

*** *p* < .001.

#### Gender composition

Gender composition was examined in two ways; after same-gender and opposite-gender dyads were compared, all four possible gender compositions—older girl–younger girl, older girl–younger boy, older boy–younger boy, and older boy–younger girl—were explored. Preliminary analyses showed no differences between the groups in ratio of boys to girls, firstborn mean age, sociodemographic risk, verbal IQ, and working memory or in inhibition across all of the groups (all *p*s > .10). No associations were detected between gender compositions of sibling dyads and second-order false belief (*p*s > .20).

#### Birth interval

**Fig. 5:**
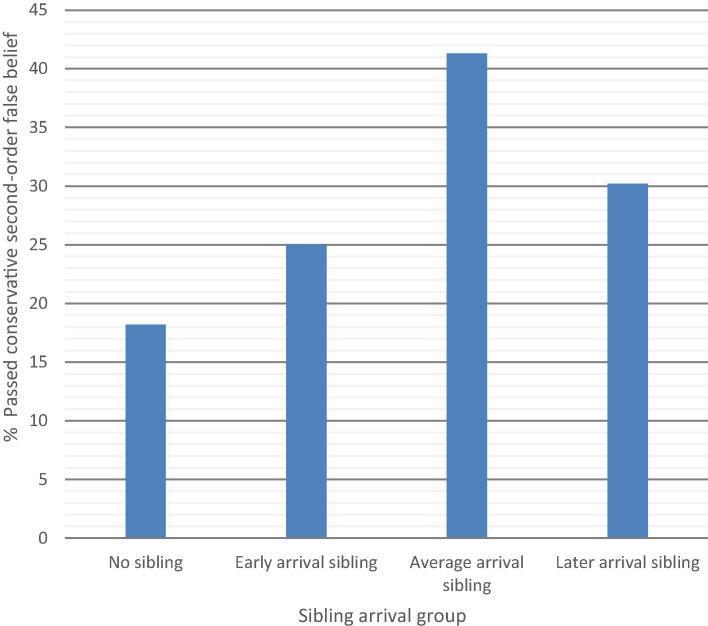
Percentages of children who passed the second-order false belief task with full comprehension according to sibling arrival groups.

Although no association was detected between timing of sibling arrival and second-order false belief understanding (*r* = .02, *p* = .81) (see Table 2), the four sibling arrival groups (no-sibling group and early-, average-, and late-arriving sibling groups) were investigated. Preliminary analyses showed no differences among these groups in ratio of boys to girls, verbal IQ, and working memory (*p*s > .15). Significant differences were detected among groups in sibling age, *F*(3, 224) = 3.16, *p* < .05, sociodemographic risk, *F*(3, 225) = 4.13, *p* < .01, and ANT inhibition scores, *F*(3, 220) = 3.12, *p* < .05. Post hoc tests were selected in accordance with results from tests for homogeneity of variances. Games–Howell post hoc tests indicated that children in the late-arriving sibling group were older than those in the no-sibling group and that children with an average-arriving sibling performed better on the inhibition task than those without a sibling (*p*s < .05). A Tukey post hoc test indicated that children with an average-arriving younger sibling had lower sociodemographic risk than those with a late-arriving sibling (*p* < .01). A significant difference was detected among the four sibling groups in their passing of the second-order false belief task with full comprehension, *χ*^2^(3) = 8.97, *p* < .05 (Fig. 5).

This finding was explored further while controlling for covariates of second-order false belief. Because late-arriving siblings did not significantly differ from the average-arriving sibling group in performance on passing second-order false belief with full comprehension, these were collapsed into one “average to late”-arriving sibling group. There were no significant differences among the groups in ratio of boys to girls, firstborn mean age, sociodemographic risk, verbal IQ, and working memory or in inhibition when these groups were collapsed (all *p*s > .06).

The three remaining sibling status groups were dummy coded, with the no-sibling group assigned as the reference category in a logistic regression. Covariates (age and verbal IQ) were entered into the first step of the logistic regression model. When entered into the model at the second step, early arrival of a younger sibling and average to later arrival of a younger sibling accounted for a significant step when entered into the model, accounting for an additional 4% of the variance in second-order false belief with full comprehension, *χ*^2^(2) = 6.57, *p* < .05. The overall model remained significant, *χ*^2^(2) = 21.57, *p* < .001, Nagelkerke *R*^2^ = .13. The early arrival of younger siblings did not predict firstborns’ passing of second-order false belief with full comprehension; however, “average to late”-arriving siblings conveyed a significant advantage, Wald statistic = 5.63, *p* < .05, *OR* = 2.66, 95% *CI* = 1.19–5.96 (Table 5).

**Table 5:** Logistic regression of dummy-coded sibling status groups, firstborn age, and verbal IQ as predictors of passing second-order false belief with full comprehension.

Variable	*R*^2^	*B*	*SE*	Wald *χ*^2^	OR (Odds Ratio)	95% *CI* for *OR*
Step 1	.09***					
Constant		−10.91	3.61	9.12	0.00	
Firstborn age		.06	.04	2.86	1.06	0.99–1.14
Verbal IQ		.05***	.01	12.68	1.05	1.02–1.08

Step 2	.13***					
Constant		−10.87	3.71	8.58	0.00	
Firstborn age		.05	.04	1.86	1.05	0.98–1.13
Verbal IQ		.05	.02	12.87	1.05	1.02–1.08
Early arrival younger sibling		.46	.51	0.82	1.59	0.58–4.34
Average to late arrival younger sibling		.98*	.41	5.63	2.66	1.19–5.96

*Note.* The table presents the total *R*^2^ Nagelkerke statistic. *N* = 219.

* *p* < .05.

*** *p* < .001.

## Discussion

When predictors of second-order false belief understanding were controlled, children with a younger sibling living in the home were twice as likely to succeed on a second-order false belief task. It was established that this sibling advantage occurred only for firstborns who did not experience the early arrival of a sibling. Our finding stands in contrast to the first study of sibling effects on second-order false belief tasks, which found no effect (Miller, 2013), but is consistent with previous research showing that presence of a younger sibling in the home is advantageous for ToM (Lewis et al., 1996, Perner et al., 1994, Peterson, 2000). In contrast to earlier work (Kennedy et al., 2015), the younger sibling’s influence on a higher-order ToM task in our sample was not limited to same-sex siblings.

There are various mechanisms by which younger siblings could facilitate their siblings’ social understanding; these might include engaging in joint pretense (Youngblade & Dunn, 1995), sharing knowledge through teaching (Azmitia and Hesser, 1993, Zajonc and Markus, 1975), or engaging in conflict and resolution (Dunn, 1994, Foote and Holmes-Lonergan, 2003). Our focus on younger siblings, however, revealed that the firstborn’s experience of the arrival of a younger sibling before the second birthday did not provide a similar advantage. The first 2 years of life represent an important time in ToM development, when evidence for consciousness, pretense, and the use of lexical terms for mental states emerges (Astington et al., 1988, Bartsch and Wellman, 1995). Transition to siblinghood during this time may disrupt this process. Future work should examine multiple factors in family interactions that explain differential effects of early- and late-arriving siblings on the oldest child’s social cognitive development.

Children who experienced socioeconomic adversity performed less well on the second-order task; however, this association did not remain significant when accounting for age and verbal IQ. This finding stands in contrast to previous research (Cole and Mitchell, 2000, Cutting and Dunn, 1999), perhaps because our study took into account a number of dimensions of sociodemographic risk beyond occupational class or income. Although a number of sociodemographic risk factors have been found to be associated with ToM, such as income, maternal education (Andersson, Sommerfelt, Sonnander, & Ahlsten, 1996), and parental occupational class (Cutting & Dunn, 1999), rarely are these factors all controlled in a single study (Pears & Moses, 2003).

Although the effects reported in this study were not large, it is important to note that the sample size used in the current study provided sufficient power to enable detection of such small to moderate effects. Thus, the absence of an association with children’s executive function abilities in this sample is noteworthy given that there was sufficient power to detect such an effect. Although executive function abilities and first-order ToM have been found to be positively related (Carlson, Moses, & Breton, 2002), a finding replicated in the current study with respect to working memory in particular, there has not been consistent evidence for a correlation between executive function and second-order ToM (for a review, see Miller, 2009). Indeed, executive function has been found to be positively associated with second-order false belief when age was controlled, but not when language ability was controlled (Hasselhorn et al., 2005). Alternatively, it is possible that the nonverbal measures used in this study to assess executive function might not be comparable to other verbal measures of inhibition and working memory such as Bear/Dragon, “Simon Says”–type inhibition tasks or word/digit span working memory tasks (Carlson et al., 2002). Before a more definitive conclusion can be made, replication of this finding using other executive function tasks is warranted.

In light of previous research suggesting that some 6-year-olds and the majority of 7-year-olds are successful at attributing second-order beliefs (Perner & Wimmer, 1985), it is noteworthy that only a minority of children in this community sample passed the second-order task. This finding must be interpreted with some caution in view of the limitations of our study procedures. Data collection took place in the family homes; therefore the assessment may have been influenced by distractions within the home environment. However, evidence from this representative community sample may provide a more accurate estimate of the number of children at this age who understand second-order false belief. Finally, given our sampling strategy where we recruited firstborn children, we are unable to determine whether our findings were driven by a general sibling effect, not just the influence of younger siblings. Therefore, more work is needed to determine whether older siblings, as well as younger siblings, continue to foster children’s understanding of minds into middle childhood.

In conclusion, the finding that the presence of a younger sibling in the home facilitated the firstborn’s false belief understanding draws attention to the unique contribution of the sibling relationship to social cognitive development during middle childhood. Taken together with evidence from the vast literature on first-order false belief understanding, our findings contribute to knowledge about the influence of both younger and older siblings on a child’s development of a ToM during the middle childhood years.
